# Age-Related Changes of Plasma Bile Acid Concentrations in Healthy Adults—Results from the Cross-Sectional KarMeN Study

**DOI:** 10.1371/journal.pone.0153959

**Published:** 2016-04-19

**Authors:** Lara Frommherz, Achim Bub, Eva Hummel, Manuela J. Rist, Alexander Roth, Bernhard Watzl, Sabine E. Kulling

**Affiliations:** 1 Department of Safety and Quality of Fruit and Vegetables, Max Rubner-Institut, Federal Research Institute of Nutrition and Food, Karlsruhe, Germany; 2 Department of Physiology and Biochemistry of Nutrition, Max Rubner-Institut, Federal Research Institute of Nutrition and Food, Karlsruhe, Germany; 3 Department of Nutritional Behaviour, Max Rubner-Institut, Federal Research Institute of Nutrition and Food, Karlsruhe, Germany; University of Insubria, ITALY

## Abstract

Bile acids (BA) play an important role in lipid metabolism. They facilitate intestinal lipid absorption, and BA synthesis is the main catabolic pathway for cholesterol. The objective of this study was to investigate associations of age, sex, diet (fat intake) and parameters of lipid metabolism (triglycerides, LDL, HDL, body fat content) with fasting plasma BA concentration of healthy individuals. Fasting plasma samples from a cross-sectional study were used to determine the concentrations of 14 BA using an LC-MS stable isotope dilution assay. Triglycerides, LDL and HDL were analyzed by standard clinical chemistry methods and body fat content was measured with a DXA instrument. The dietary fat intake of the 24 h period prior to the sampling was assessed on the basis of a 24 h recall. Subsequent statistical data processing was done by means of a median regression model. Results revealed large inter-individual variations. Overall, higher median plasma concentrations of BA were observed in men than in women. Quantile regression showed significant interactions of selected BA with age and sex, affecting primarily chenodeoxycholic acid and its conjugates. No associations were found for LDL and the amount of fat intake (based on the percentage of energy intake from dietary fat as well as total fat intake). Additional associations regarding body fat content, HDL and triglycerides were found for some secondary BA plasma concentrations. We conclude that age and sex are associated with the fasting plasma concentrations. Those associations are significant and need to be considered in studies investigating the role of BA in the human metabolism.

## Background

BA are synthesized in the liver from cholesterol, conjugated to taurine or glycine and stored in the gallbladder. Upon food intake the small intestine secretes cholecystokinin (CCK) which stimulates the postprandial contraction of the gallbladder. BA are then excreted via the bile duct into the small intestine and partially transformed into various types of secondary BA by the intestinal microbiota. They are subjected to enterohepatic circulation by active and passive absorption and are transported in the blood with an affinity to bind to serum proteins (mainly albumin) and lipoproteins depending on their hydrophobicity [1–3]. Incomplete hepatic recovery of BA from the portal vein seems to result in low concentrations of BA in the peripheral circulation [4, 5].

As has been known for many decades, BA are an essential part of bile and are important for multiple physiological functions in the gastrointestinal tract including the absorption of lipophilic nutrients and the inhibition of bacterial overgrowth in the small intestine [6]. Only in the last 10 years, it has been found that BA in the peripheral blood also have regulatory functions in the carbohydrate, lipid and energy metabolism by binding to nuclear receptors like the farnesoid x receptor (FXR) and the G-protein coupled receptor TGR5 [4]. As such they are investigated in the context of diseases such as metabolic syndrome and diabetes mellitus type 2 [7, 8]. For example, fasting plasma concentrations of some selected BA have been found to be inversely correlated with insulin sensitivity of adults [9].

BA metabolites and BA profiles in plasma of healthy people have been well characterized in recent studies [10–14]. However, the results in these studies are not stratified and evaluated in regards to age or sex. Those studies which did account for age or sex in their statistical evaluation, report about differences in BA plasma concentrations between men and women [1, 15, 16], and associations with age [17], but not the interaction between age and sex.

The aim of our study was to comprehensively characterize BA plasma concentrations for healthy males and females, respectively, in relation to their age. We further wanted to know whether the intake of fat (based on the percentage of energy intake from dietary fat (Energy Fat%) and total fat intake) as well as parameters of lipid metabolism, namely triglycerides (TG), LDL, HDL, body fat content (BF%) correlate with those plasma concentrations. We used fasting plasma samples from 300 healthy participants of the cross-sectional KarMeN (Karlsruhe Metabolomics and Nutrition) study.

## Materials and Methods

### Participants and study design

KarMeN (Karlsruhe Metabolomics and Nutrition) is a cross-sectional study designed to investigate the human metabolome in blood and urine and its determinants in healthy participants. Additionally, we aimed to determine the role of sex, age and body composition, as well as the role of the major lifestyle factors diet and physical activity on metabolite profiles of healthy adults. Participants were screened between May 2012 and August 2013 and included in the study based on the following criteria: healthy and no history of a chronic disease, non-smokers, no medication or intake of any kind of hormones or supplements. To ensure that results were not distorted by participants with an altered metabolism, data gained from participants during the study was evaluated for any possible health problems and anomalies and excluded, if that was the case. The study was registered at the German Clinical Trials Register (No. DRKS 00004890) and approved by the ethics committee of the State Medical Chamber, Baden Württemberg (F-2011-051). Written consent was obtained from all participants. A comprehensive set of anthropometric, medical, and life-style data from 300 participants (172 male, 128 female; BMI 17.8–31.4 kg/m^2^; age: 18–80 y) were assessed and blood as well as urine samples were taken. Blood samples taken between 7–9 am after an over-night fasting period were used to determine plasma BA concentrations according to the method described in this manuscript.

Body composition including body fat content (BF%) and body fat distribution was measured using a Lunar iDXA-instrument (General Electric). For female participants, postmenopausal status of women not on a regular menstrual cycle was determined by respective anamnestic interview and Follicle Stimulating Hormone (FSH) measurements. We assumed postmenopausal status by absence of menstrual bleeding for at least one year and FSH > 25 IU/L. Food consumption of the day prior to blood sample collection was assessed by the 24-h recall method using the software EPIC-Soft® [18]. Energy Fat% was calculated based on the German Nutrient Database (BLS) version 3.02 [19].

### Clinical Chemistry

TG, HDL, LDL, serum glucose and FSH were measured by MVZ Labor PD Dr. Volkmann, Karlsruhe, Germany. TG, HDL, LDL and serum glucose were determined with an enzymatic, colorimetric method on an automated modular blood analyzer system (Roche cobas 8000). The precision of these methods in terms of repeatability are 0.6–0.8 (CV%) for HDL, 0.5–0.9 (CV%) for LDL, 0.5–0.8 (CV%) for serum glucose and 0.6–0.9 (CV%) for TG. Follicle Stimulating Hormone was determined by an immunoluminometric assay.

### Chemicals

Cholic acid (CA), deoxycholic acid (DCA), lithocholic acid (LCA), chenodeoxycholic acid (CDCA), taurolithocholic acid (TLCA), ursodeoxycholic acid (UDCA), taurochenodeoxycholic acid (TCDCA), glycocholic acid (GCA), glycochenodeoxycholic acid (GCDCA), taurodeoxycholic acid (TDCA) and taurocholic acid (TCA) were purchased from Sigma-Aldrich (Steinheim, Germany). Glycoursodeoxycholic acid (GUDCA), tauroursodeoxycholic acid (TUDCA) and glycodeoxycholic acid (GDCA) were from Calbiochem (La Jolla, USA). Deuterated internal standards (IS) deoxycholic-2,2,4,4-d4 acid (DCA-d4), glycoursodeoxycholic-2,2,4,4-d4 acid (GUDCA-d4), glycodeoxycholic-2,2,4,4-d4 acid (GDCA-d4), taurocholic-2,2,3,4,4-d5 acid (TCA-d5) were obtained from CDN Isotopes (Pointe-Claire, Canada) and glycocholic-2,2,4,5-d4 acid (GCA-d4), cholic-2,2,4,4-d4 Acid (CA-d4) from Campro Scientific (Berlin, Germany). HPLC-grade methanol (MeOH), acetonitrile and formic acid were purchased from VWR (Darmstadt, Germany).

### HPLC-MS analysis

Chromatographic separation was achieved on an 1100 Series HPLC (Agilent, Waldbronn, Germany) equipped with a Phenomenex Luna C18 (150 x 3 mm, 3 μm) column and corresponding pre-column (4 x 3 mm). The column temperature was 40°C. The HPLC mobile phases consisted of 5 mM aqueous ammonium acetate, adjusted to a pH of 5.2 with approx. 0.005% formic acid (A) and acetonitrile (B). The following linear gradient with a flow rate of 0.6 ml/min was used (% B): 0–1 min (35%), 9–11 min (70%), 12–16 min (95%), and 17–22 min (35%).

The HPLC system was coupled to a 3200 QTrap mass spectrometer (ABSciex, Darmstadt, Germany). Electrospray ionization was performed in the negative mode using the following parameters: 40 psi (curtain gas), 600°C (Source Temperature), -4500 V (Ion Spray Voltage), 50 psi/60 psi (Ion Gas 1 and 2, respectively). Data were recorded in the multiple reaction monitoring mode (MRM) with nitrogen as a collision gas. System operation, data acquisition and subsequent quantification were achieved by using Analyst 1.5.2. software (AB Sciex) and Multiquant 2.1.1. (AB Sciex).

Declustering potential, collision cell parameters and transitions were optimized for each compound (S1: Table).

### Preparation of Standard Solutions and Calibration Curves

Stock solutions of the individual BA standards and the deuterated IS were prepared at a concentration of 10 mM in MeOH. Mixed stock solutions (one for BA standards and one for the deuterated IS, 100 μM for each compound) were prepared in MeOH. These mixed stock solutions were further diluted with MeOH:H_2_O (50:50, v:v) to obtain standard working solutions (10 μM, 1 μM, 100 nM) and an IS working solution (1 μM). Pooled plasma samples were stripped with activated charcoal according to Steiner et al [20]. Calibration samples were prepared by adding an appropriate amount of BA standard working solution to 100 μl of stripped plasma to obtain BA concentrations of 25, 100, 1000 and 2500 nM, respectively.

### Sample Preparation and SPE procedure

Plasma samples were stored at -80°C until analysis and were allowed to thaw on ice. 100 μl of IS working solution and 700 μl of 0.005% formic acid were added to 100 μl plasma sample and to each stripped plasma calibrator. The entire sample was transferred onto SPE columns (Strata X, 30 mg, Phenomenex) which were conditioned with 1 ml MeOH and 1 ml 0.005% formic acid. Samples were washed with 1 ml H_2_O and 1 ml 5% MeOH and dried under vacuum for 10 min. BA were eluted with 1 ml MeOH and 1 ml acetonitrile. Eluents were evaporated to dryness under a stream of nitrogen at room temperature, reconstituted with 50 μl MeOH:H_2_O (50:50, v:v) and centrifuged for 5 min at 3000 rpm. 10 μl of the supernatant were used for HPLC-MS analysis.

### Method Validation

Method validation was done according to FDA guidelines for bioanalytical method validation [21] determining the level of detection (LOD), the level of quantitation (LOQ), precision and accuracy (for 25 nM, 100 nM and 1000 nM (n = 6)). Recovery and matrix effects were investigated according to Matuszewski et al. [22] using stripped plasma for assessment (n = 5). To ensure precision in between batches, pooled plasma samples from the study were used as quality control sample and measured three times within an extraction batch. A summary of the validation parameters can be found in S2A–S2C Table.

### Statistical Approach

A linear median regression approach was chosen because the distributions of the BA were substantially skewed [23]. BA with more than 25% of the values below LOD were not considered for further analysis by means of median regression. For the remaining BA, values below LOD were set to LOD/2.

All calculations were carried out using R 3.1.2 [24]. For quantile regression the package “quantreg” version 5.11 was used [25].

#### Models including BA profile and age

The subject dependent BA profile was created by dividing the respective absolute BA concentration by the total sum of all BA. These relative concentrations sum up to 1 for each subject. This approach allows investigating if the relative BA composition changes with age. For males four age groups were defined (18–35 years, 36–50 years, 51–65 years and 66–60 years). For females instead of age, menopause status was used to define groups.

Because the relative concentrations are bounded between 0 and 1, a mixed beta regression model was applied with the relative concentrations as dependent variable and age group/menopause status and the respective BA as independent categorical variables. SAS procedure PROC GLIMMIX was used to calculate the model parameters.

#### Models including age and sex

The associations between median BA concentrations and the independent factors sex and age were investigated by means of a median regression model. Age was entered as a continuous variable into the model. In order to improve interpretation of the intercept (β_0_), age was centered by subtracting the mean age of every subject. Hence, the intercept is the estimated median BA concentration for a middle-aged man.

In addition to the two main effects age (β_1_) and sex (β_2_), an interaction term sex*age (β_4_) was introduced. Besides estimates that are based on the original scale of the included variables, standardized estimates were added to the result tables. Standardized estimates were calculated by transforming all continuous variables (mean centered and scaled by one standard deviation). This allows comparing the effects of variables that were measured on different scales, because the estimates of the corresponding standardized ß represent the change of the dependent variable when the independent variable is increased by a unit change (one standard deviation). Formally, the model was specified according to the following equation:

$$BA=\beta_{0}+\beta_{1}\cdot Age+\beta_{2}\cdot Sex+\beta_{4}\cdot Age\cdot Sex$$

#### Models including age, sex and one lipid metabolism parameter

Parameters of lipid metabolism (TG, LDL, HDL, BF%, Energy Fat%, total fat intake) were consecutively taken into the model described above to account for their possible associations with BA. In accordance with the continuous variable age, continuous lipid metabolism parameters were centered as well. In this case the intercept represents the estimated median BA concentration for a middle aged man with a mean lipid metabolism parameter.

β_3_ is the main effect for the lipid metabolism parameter. β_5_ describes the two-way interaction between age and a respective lipid metabolism parameter, while β_6_ defines the interaction between sex and a lipid metabolism parameter. Finally, β_7_ denotes the three-way interaction between age, sex and a lipid metabolism parameter. Formally, the model was specified according to the following equation:

$$\text{BA}=\beta_{0}+\beta_{1}\cdot \text{Age}+\beta_{2}\cdot \mathrm{Sex}+\beta_{3}\cdot \text{PLM}+\beta_{4}\cdot \mathrm{Sex}\cdot \text{Age}+\beta_{5}\cdot \text{Age}\cdot \text{PLM}+\beta_{6}\cdot \mathrm{Sex}\cdot \text{PLM}+\beta_{7}\cdot \text{Age}\cdot \mathrm{Sex}\cdot \text{PLM}$$

with PLM denoting one parameter of lipid metabolism.

## Results

### BA concentrations in study samples

Fasting plasma concentrations showed large variations between the different BA as well as between individuals (Table 1). Low concentrations of taurine-conjugated species resulted in a large number of values below LOD. BA detected in less than 75% of the samples were not included in subsequent statistical evaluation. This was the case for TUDCA and TLCA. For statistical analysis, BA were initially grouped into primary, secondary, glycine- and taurine-conjugated BA. However, significant results were observed with individual BA rather than among the groups. Consequently, subsequent analyses were done with individual BA data.

Fasting plasma concentrations of individual BA in men all decreased with age. Most BA concentrations in women remained unaffected (e.g. GCDCA) or slightly increased with age (e.g. CDCA, CA) as indicated by the Spearman correlation in Table 1 and supported by results of the quantile regression (see below). Overall, the median plasma concentrations of BA were higher in men than in women.

**Table 1:** Results of participants fasting plasma BA concentrations (nM) to display their inter-individual variability.

	Male n = 172						Female n = 128						All n = 300					
BA (nM)	Median	Q1	Q3	Min	Max	Spearman Cor.	Median	Q1	Q3	Min	Max	Spearman Cor.	Median	Q1	Q3	Min	Max	Spearman Cor.
**GCDCA**	785	422	1497	61.1	4427	-0.343	576	288	893	64.1	6802	-0.008	669	373	1140	61.1	6802	-0.247
**DCA**	391	228	642	7.0	2678	-0.244	291	149	451	2.5 ^**a**^	2570	-0.079	351	188	566	2.5 ^**a**^	2678	-0.224
**GDCA**	253	124	439	6.6	2260	-0.300	178	76.9	351	2.5 ^**a**^	3209	-0.103	222	99.7	412	2.5 ^**a**^	3209	-0.255
**GCA**	255	142	372	15.0	1540	-0.284	151	74.3	270	14.2	6573	-0.099	204	105	334	14.2	6573	-0.251
**CDCA**	259	116	543	11.0	6773	-0.237	124	49.4	282	15.0	5415	0.093	188	81.6	431	11.0	6773	-0.154
**CA**	111	44.6	406	2.5^**a**^	6813	-0.032	66.9	28.0	208	8.4	5594	0.090	83.6	35.0	330	2.5 ^**a**^	6813	-0.031
**GUDCA**	102	55.2	221	9.6	940	-0.306	54.1	27.0	135	7.3	702	-0.024	76.6	43.4	178	7.3	940	-0.238
**UDCA**	73.7	40.8	135	11.4	694	-0.340	61.6	36.9	105	9.1	1195	-0.101	69.7	38.5	119	9.1	1195	-0.266
**TCDCA**	57.3	26.9	111	2.5 ^**a**^	729	-0.297	52.7	26.7	96.2	2.5 ^**a**^	1970	-0.024	55.4	26.9	102	2.5 ^**a**^	1970	-0.202
**TDCA**	36.9	16.0	67.5	2.5 ^**a**^	385	-0.241	29.5	14.1	61.9	2.5 ^**a**^	1719	-0.114	33.9	15.0	64.3	2.5 ^**a**^	1719	-0.209
**LCA**	21.8	15.6	31.0	2.5 ^**a**^	104	-0.049	23.7	18.0	35.4	6.3	99.2	0.111	23.0	16.0	33.6	2.5 ^**a**^	104	0.037
**TCA**	20.5	8.9	42.7	2.5 ^**a**^	539	-0.248	15.8	6.7	33.2	2.5 ^**a**^	2166	-0.110	18.0	8.0	38.9	2.5 ^**a**^	2166	-0.211
**total BA**	2686	1695	5005	566	20130	-0.317	1928	1151	3134	411	18289	-0.019	2232	1552	4375	411	20130	-0.244

Fasting plasma BA concentrations of participants are stratified by sex, displaying the median fasting plasma concentration, the 25^th^ percentile (Q1), the 75^th^ percentile (Q3), the minimum (Min) and maximum (Max) concentration for each BA. Correlation of data with age is indicated by Spearman correlation (r_S_).

^a^: Values below LOD (<5nM) were set to LOD/2, resulting in minimum concentrations of 2.5 nM.

### Mixed beta regression (BA profile and age)

Results from the mixed beta regression model regarding the BA profiles did not reveal any association with age for men, when age was used as categorical variable. Also, no difference in the BA profile between pre- and postmenopausal women was found. Illustrations of BA profiles can be found in S1A and S1B Fig.

### Quantile regression (age and sex)

A quantile regression was performed in order to investigate the association of age and sex with BA concentrations in more detail. Results revealed significant interactions between age and sex concerning the concentration of CDCA, GCDCA, TCDCA, and GUDCA (p-values ß_4_, Table 2). This clearly indicates that for these BA the age-dependent change in concentrations differs between men and women.

**Table 2:** Results of quantile regression analysis of individual bile acid concentrations with sex and age.

BA	ß_0_	ß_1_	ß_2_	ß_4_
**GCDCA estimates**	**733.653**	**-15.673**	**-167.252**	**15.121**
**GCDCA std. estimates**	**-0.131**	**-0.266**	**-0.083**	**0.256**
**GCDCA p-values**	**<0.001**	**<0.001**	**0.042**	**<0.001**
**DCA estimates**	**397.511**	**-4.652**	**-89.601**	2.580
**DCA std. estimates**	**-0.078**	**-0.187**	**-0.105**	0.104
**DCA p-values**	**<0.001**	**0.003**	**0.036**	0.260
**GDCA estimates**	**259.938**	**-4.741**	-63.131	3.112
**GDCA std. estimates**	**-0.093**	**-0.215**	-0.084	0.141
**GDCA p-values**	**<0.001**	**<0.001**	0.095	0.195
**GCA estimates**	**243.967**	**-2.835**	**-91.540**	2.483
**GCA std. estimates**	**-0.078**	**-0.080**	**-0.076**	0.070
**GCA p-values**	**<0.001**	**0.001**	**<0.001**	0.091
**CDCA estimates**	**236.890**	**-4.393**	**-112.151**	**6.405**
**CDCA std. estimates**	**-0.129**	**-0.105**	**-0.078**	**0.153**
**CDCA p-values**	**<0.001**	**<0.001**	**<0.001**	**<0.001**
**CA estimates**	**108.157**	-0.201	-46.126	1.005
**CA std. estimates**	**-0.158**	-0.005	-0.033	0.024
**CA p-values**	**<0.001**	0.891	0.072	0.516
**GUDCA estimates**	**103.973**	**-1.934**	**-48.005**	**1.775**
**GUDCA std. estimates**	**-0.115**	**-0.205**	**-0.149**	**0.188**
**GUDCA p-values**	**<0.001**	**<0.001**	**0.001**	**0.006**
**UDCA estimates**	**78.740**	**-1.363**	**-17.683**	1.123
**UDCA std. estimates**	**-0.103**	**-0.185**	**-0.070**	0.152
**UDCA p-values**	**<0.001**	**<0.001**	**0.010**	0.060
**TCDCA estimates**	**56.082**	**-1.142**	-1.365	**0.963**
**TCDCA std. estimates**	**-0.122**	**-0.103**	-0.004	**0.087**
**TCDCA p-values**	**<0.001**	**<0.001**	0.844	**0.021**
**TDCA estimates**	**33.598**	**-0.664**	-0.491	0.117
**TDCA std. estimates**	**-0.107**	**-0.097**	-0.002	0.017
**TDCA p-values**	**<0.001**	**<0.001**	0.922	0.702
**LCA estimates**	**21.668**	-0.071	0.898	0.218
**LCA std. estimates**	**-0.145**	-0.079	0.029	0.243
**LCA p-values**	**<0.001**	0.283	0.662	0.083
**TCA estimates**	**19.765**	**-0.400**	-4.192	0.309
**TCA std. estimates**	**-0.098**	**-0.037**	-0.011	0.029
**TCA p-values**	**<0.001**	**<0.001**	0.104	0.063
**total BA estimates**	**2886.5**	**-48.000**	**-866.99**	**37.445**
**total BA std. estimates**	**-0.082**	**-0.272**	**-0.144**	**0.212**
total BA p-values	**<0.001**	**<0.001**	**0.001**	**0.014**

Rows 1–3 denote estimates, standardized estimates and p-values (rows 1–3 for each BA, respectively) for beta coefficients of the quantile regression (sex and age). Significant results (P < 0.05) are in bold print.

**Fig 1:**
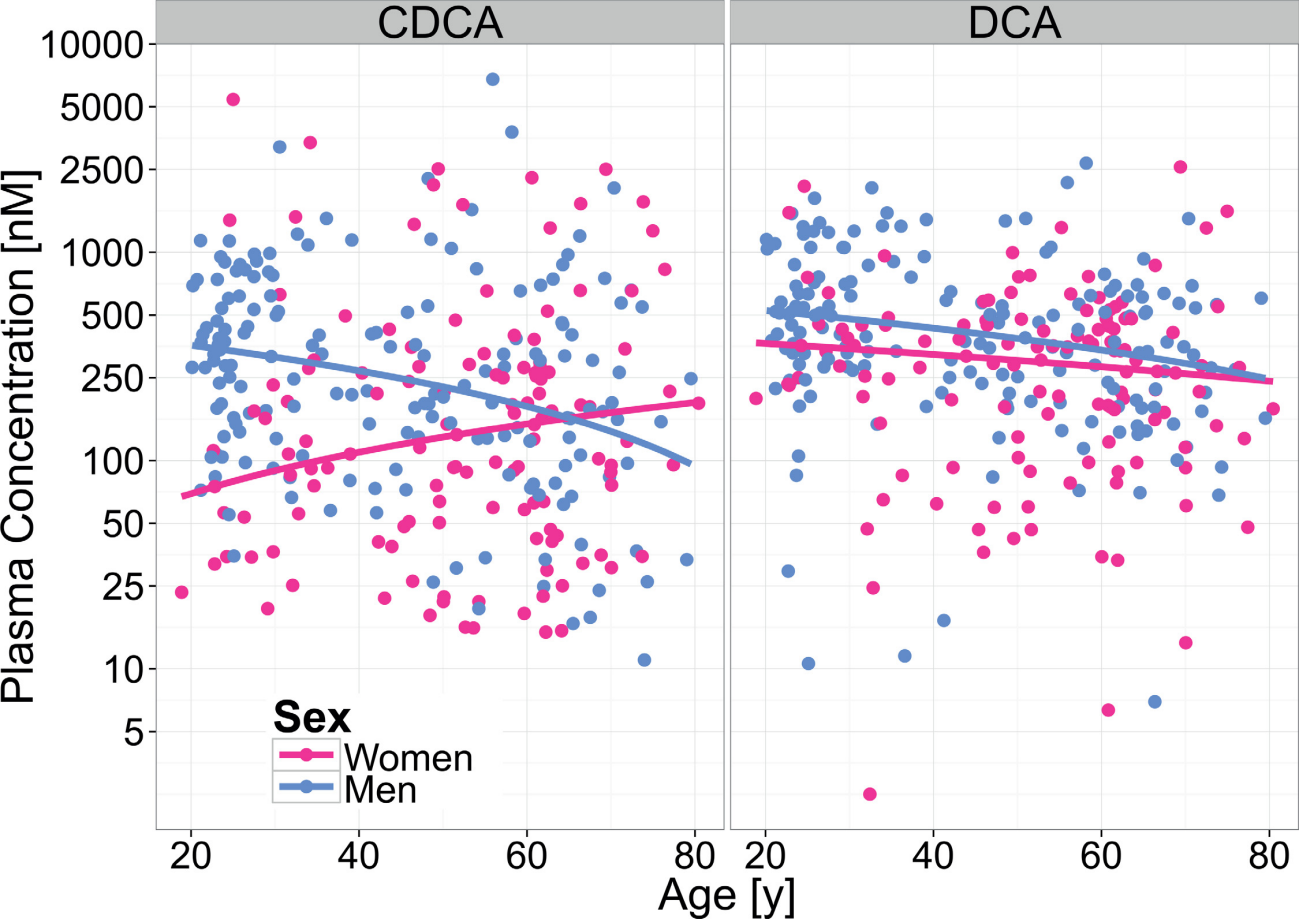
Sex- and age-dependency of CDCA and DCA in fasting plasma. Selected plot demonstrating the interaction of sex and age (CDCA), and an equal effect of age in both sexes (DCA) regarding the fasting plasma concentrations. Lines depict the predicted values according to the median regression model for men (blue) and women (pink).

No interactions were observed for the plasma concentrations for DCA, GCA, UDCA, GDCA and TDCA (p-values ß_4_, Table 2). The concentrations of these BA decreased with age (p-values ß_1_, Table 2) in both sexes. The median concentrations of DCA, GCA and UDCA were significantly different between men and women; this was not true for GDCA and TDCA (p-values ß_2_, Table 2). We did not find any differences in plasma concentrations of CA, LCA, TUDCA and TLCA between men and women, nor did we observe any association with age. Selected plots from the quantile regression (age and sex) are displayed in Fig 1; plots for the remaining BA are shown in S2 Fig.

### Quantile regression (age, sex, lipid metabolism parameters)

BA play a major role in lipid absorption and metabolism, therefore other parameters associated with lipid metabolism, including TG, LDL, HDL, BF%, Energy Fat% and total fat intake (Table 3) were explored upon their possible association with BA plasma concentrations. Results for Energy Fat% and total fat intake were almost identical. We will hence refer to results from Energy Fat% only.

**Table 3:** Anthropometric and clinical data of the participants stratified by sex.

	Male n = 172							Female n = 128						All n = 300					
	Median		Q1	Q3	Min	Max	Spearman Cor.	Median	Q1	Q3	Min	Max	Spearman Cor.	Median	Q1	Q3	Min	Max	Spearman Cor.
**age (years)**	44	26		62	20	80		54	41	62	19	80		49	30	62	19	80	
**BMI(kg/m**^**2**^**)**	24.0	22.4		26.6	19.0	31.4		22.7	20.9	24.8	17.8	30.9		23.7	21.9	25.9	17.8	31.4	
**TG (mg/dl)**	88	63		124	29	316	0.315	76	56	94	33	280	0.360	82	60	111	29	316	0.291
**HDL (mg/dl)**	62	52		70	30	102	0.058	77	67	87	39	130	0.031	67	57	79	30	130	0.139
**LDL (mg/dl)**	119	89		150	49	240	0.590	126	102	161	66	233	0.546	122	94	155	49	240	0.570
**Body Fat (%)**	23.6	17.7		27.7	8.5	36.7	0.506	34.1	28.5	38.2	18.0	47.9	0.414	27.3	21.9	33.4	8.5	47.9	0.468
**Energy Fat (%)**	37.3	30.7		44.0	11.4	70.0	-0.060	38.1	32.0	44.0	12.3	61.8	0.065	37.8	31.6	44.0	11.4	70.0	-0.011

Data presented is displaying the respective median values, the 25^th^ percentile (Q1), the 75^th^ percentile (Q3), the minimum (Min) and maximum (Max) values. Correlation of data with age is indicated by Spearman correlation (r_S_).

Plasma concentrations of TG, LDL, HDL and BF% strongly depended on age and/or sex, whereas Energy Fat% had no association with sex or age (S3 Fig). In order to investigate the relationship between these parameters and BA plasma concentrations, the subsequent statistical approach (quantile regression) accounted for sex and age. This approach resulted in an additional statistical model for each lipid metabolism parameter. The detailed results containing the five models for the lipid metabolism parameters can be found in S3 Table.

The statistical model including the additional effect of TG on the BA plasma concentration revealed a significant interaction between age, sex and TDCA (p-values ß_7_, S3 Table TG). Similar significant interaction (p-values ß_7_, S3 Table HDL) could be found concerning the GDCA concentration in the model including the effect of HDL. The concentrations of TDCA and GDCA are therefore not only determined by age and sex but also by the plasma concentration of TG and HDL, respectively.

Other significant effects shown in the statistical models are a main effect of TG on GUDCA and of BF% on UDCA and LCA (p-values ß_3_, S3 Table TG and BF%). The models provide further associations on trend level (p<0.1) (S3 Table). It is noteworthy that all indicated associations were exclusively found for secondary BA.

The models including Energy Fat% and LDL revealed no significant interactions with age and sex (p values ß_5_-ß_7_, Tables Energy Fat% and LDL and no associations with BA plasma concentrations (p values ß_3_).

### Additional Parameters

Our investigation focused on associations of age, sex and lipid metabolism parameters. But as BA are not exclusively regulated by the lipid metabolism, other parameters need to be evaluated regarding their influence on BA metabolism in this study. BA have been linked to carbohydrate metabolism and some altered fasting BA concentrations are reported to be associated with insulin sensitivity [9]. For this reason we ascertained that fasting glucose levels were all within normal range (S4 Table).As there is evidence that BA composition is altered within the normal range of insulin sensitivity [26] and as fasting glucose levels of the participants increase with age, a contributing influence cannot be ruled out. Nevertheless, we were not able to reveal any associations between age, sex and fasting glucose levels on the concentration of 12α-hydroxylated BA.

Another parameter to evaluate is the intake of dietary fiber [27, 28]. The intake per kg body weight (based on 24h recall data) did not show any correlation with age or sex in our study (S4 Table). Data did also not reveal an association with primary BA.

## Discussion

Data of fasting BA plasma concentrations do not follow Gaussian frequency distribution. This has been described before by Steiner et al. [1]. We accounted for the skewed distribution by using a linear median regression model for our statistical analysis. Large inter-individual variations in fasting BA plasma concentrations observed in our study population are consistent with already published data [10, 13].

### Interaction effect between sex and age

Results from the quantile regression show a sex-dependent effect of age on some specific BA plasma concentrations (CDCA, GCDCA, TCDCA and GUDCA) which has not been reported before. The highest plasma concentrations of these BA are found in young men (age 20–35 years), and all decrease with age. The differences between sexes regarding these BA are most apparent between young men and young women, explaining the corresponding findings of Xiang et al. [15], whose study participants were exclusively in that age range.

It is remarkable that all of these BA derive from CDCA, a primary BA. In addition to being synthesized in the liver via the classical pathway, CDCA can be synthesized via an alternative pathway initiated by sterol-27-hydroxylase, a mitochondrial cytochrome P450 enzyme (CYP27A1) found in most tissues [29, 30]. CDCA can also derive from oxysterols produced via a non-specific 7-α-hydroxylase (CYP7B1) expressed in all tissues. These oxysterols are transported to the liver and subsequently converted to CDCA. The relative contributions of the alternative pathways to BA synthesis are difficult to assess and are reported to account for no more than 10% of the BA production [31, 32]. Although we have not found any descriptions of sex differences in the alternative pathways, these pathways could contribute to the observed higher CDCA and CDCA-derived BA concentrations in young men.

Knowledge of this age and sex dependency in BA plasma concentrations allows a deeper understanding of sex and age related BA associated diseases, such as the increased prevalence of gallstones in women. Young women have a higher gallstone incidence rate than young men, and this difference disappears with increasing age [33].

The female estrogen production could be one physiological explanation. Estrogens have been reported to promote biliary cholesterol secretion by enhancing the HMG-CoA reductase activity, the rate limiting enzyme in hepatic cholesterol biosynthesis. This causes a cholesterol saturation of bile and therefore a higher risk of gallstone development in women [34, 35]. At the same time estrogens have also been reported to inhibit CDCA synthesis and to lower the biliary CDCA pool [36]. CDCA, however, formerly used as a medication in the treatment of gallstone disease, can induce cholesterol gallstone dissolution and decrease cholesterol secretion [37, 38]. According to our results, young men have the highest plasma concentrations of CDCA, which may be an explanation for their low incidence rate of gallstones. Young men also have high concentrations of testosterone which decrease with age. As sex hormones have been associated with BA metabolism [39–41], we suggest high levels of testosterone as an additional explanation for the high levels of CDCA and CDCA-derived BA concentrations in young men.

### Main effect of sex

In addition to those BA whose concentrations are determined by age as well as sex (CDCA, GCDCA, TCDCA and GUDCA), we found significant sex differences in plasma concentrations of DCA, GCA and UDCA, independent of age. Plasma concentrations of these BA are significantly higher in men than in women. Similar results have been reported before [1, 15, 17, 42]. They are also supported by findings of higher BA synthesis in men based on serum levels of 7-α-hydroxy-4-cholestene-3-one (C4), an intermediate in BA synthesis [43].

Other studies report contrary findings [16, 44–48]. However, results are difficult to compare because studies differ in design regarding sex, age and health status of the participants: Some of these studies have a smaller number of participants [44, 48], possess an uneven distribution between male and female participants [16] or were aimed to reveal sex differences among babies and toddlers [45, 47] or participants with gallstone disease [46]. Also, different statistical approaches are used and some do not account for the skewed distribution of BA concentrations.

### Main effect of age

In addition to the sex-dependent age effect on BA concentrations described above, we were able to reveal sex-independent age effects regarding the plasma concentrations of GCA, DCA, GDCA and TDCA. Age-dependent plasma concentrations have been reported most recently [17]. So far other studies did not find such associations, possibly due to the exclusion of higher-aged study participants [15] or much smaller numbers of participants of all ages [1, 16].

BA synthesis is the main catabolic pathway for cholesterol. Studies exploring age-related changes in lipid metabolism suggest a decreasing BA synthesis as one possible cause for higher plasma cholesterol concentrations with increasing age. While such inverse correlations between age and BA synthesis, in support of this theory, have been revealed by some studies [49–51], others were not able to confirm such findings [43]. However, data for BA synthesis in these studies are based on BA secretion rates, biliary BA composition or plasma C4 levels as marker for BA biosynthesis. They do not report about BA levels in plasma. Our study observed that an overall decrease in BA plasma concentrations with increasing age was only true for men (Spearman Correlation Table 1). Most BA concentrations in women remained unaffected with age. Data evaluation from future research regarding this question needs to be stratified by sex.

### Effect of parameters of lipid metabolism

Results from the models investigating the additional influence of the Energy Fat% or BF% showed no association with plasma concentrations of any detected BA. These results support other findings showing that circulating BA are not linked to energy metabolism in humans [52].

Results from the remaining models investigating TG, LDL, and HDL showed no association with the plasma concentrations of any primary BA and primary BA conjugates, suggesting a minor impact of these parameters on the metabolism of primary BA.

However, associations of TG, HDL and BF% were found for secondary BA which derive from primary BA via metabolization by the intestinal microbiota. The composition of the microbiota is determined and modulated by several factors, such as the maternal gut microflora, mode of delivery, use of antibiotics [53] and diet [54, 55]. For example, the intake of a diet high in dairy-derived fat altered the composition of the gut microbiota in mice and promoted the taurine conjugation of hepatic BA [56], which means there is a strong interaction between BA and gut microbiota. In our study, we were not able to reveal any association between Energy Fat% and plasma BA concentrations. However, we did not stratify for type or origin of the dietary fats.

Another known parameter to influence BA concentration is the intake of dietary fiber. There are different proposed mechanisms by which the intake of dietary fiber is believed to influence BA concentrations. It has been shown that BA-binding properties of dietary fiber restrict the intestinal re-absorption of BA [27]. Data from recent animal research further provides alternative mechanisms by which BA concentrations may be influenced, suggesting that many variables are regulating BA metabolism [28]. However, based on our cross-sectional study data we are not able to confirm these findings, but cannot exclude dietary fiber as a possible confounder for associations of BA concentrations with the intake of lipophilic nutrients.

## Conclusions

Our data reveal associations of some BA with age or sex, as well as interactions with age and sex concerning plasma BA concentrations of CDCA and CDCA-derived BA. These associations and interactions are important for further studies investigating the role of BA in human metabolism. Other lipid metabolism parameters (TG, HDL and BF%) were associated with plasma concentrations of secondary BA. Further studies are needed to investigate the mechanistic link for these associations.

## Supporting Information

S1 Fig BA profiles of fasting plasma in men and women. (PDF)

S2 Fig Quantile Regression Plots for BA associations with age and sex. (PDF)

S3 Fig Age and Sex association of TG, LDL, HDL and BF%. (PDF)

S1 Table Instrument Settings of LC-MS methods for determination of BA. (PDF)

S2 Table Validation Parameters of LC-MS method for determination of BA. (PDF)

S3 Table Full Model Quantile Regression Results (PDF)

S4 Table Additional data of the participants stratified by sex. (DOCX)
