# Supplementary material for: Age-Related Changes of Plasma Bile Acid Concentrations in Healthy Adults—Results from the Cross-Sectional KarMeN Study
Source: PLoS One. 2016 Apr 19;11(4):e0153959. doi: 10.1371/journal.pone.0153959 (PMC4836658; doi:10.1371/journal.pone.0153959)

S1 Figure. BA Pro iles

BA profiles of men and women divided into four age groups. To improve clarity of the illustration, rather than using individual BA, the unconjugated species were grouped with their conjugates and LCA was omitted due to its low percentage. Despite the previous categorization of women into pre- and post-menopausal in the statistical models four age groups were chosen for reasons of comparability with the male profiles.

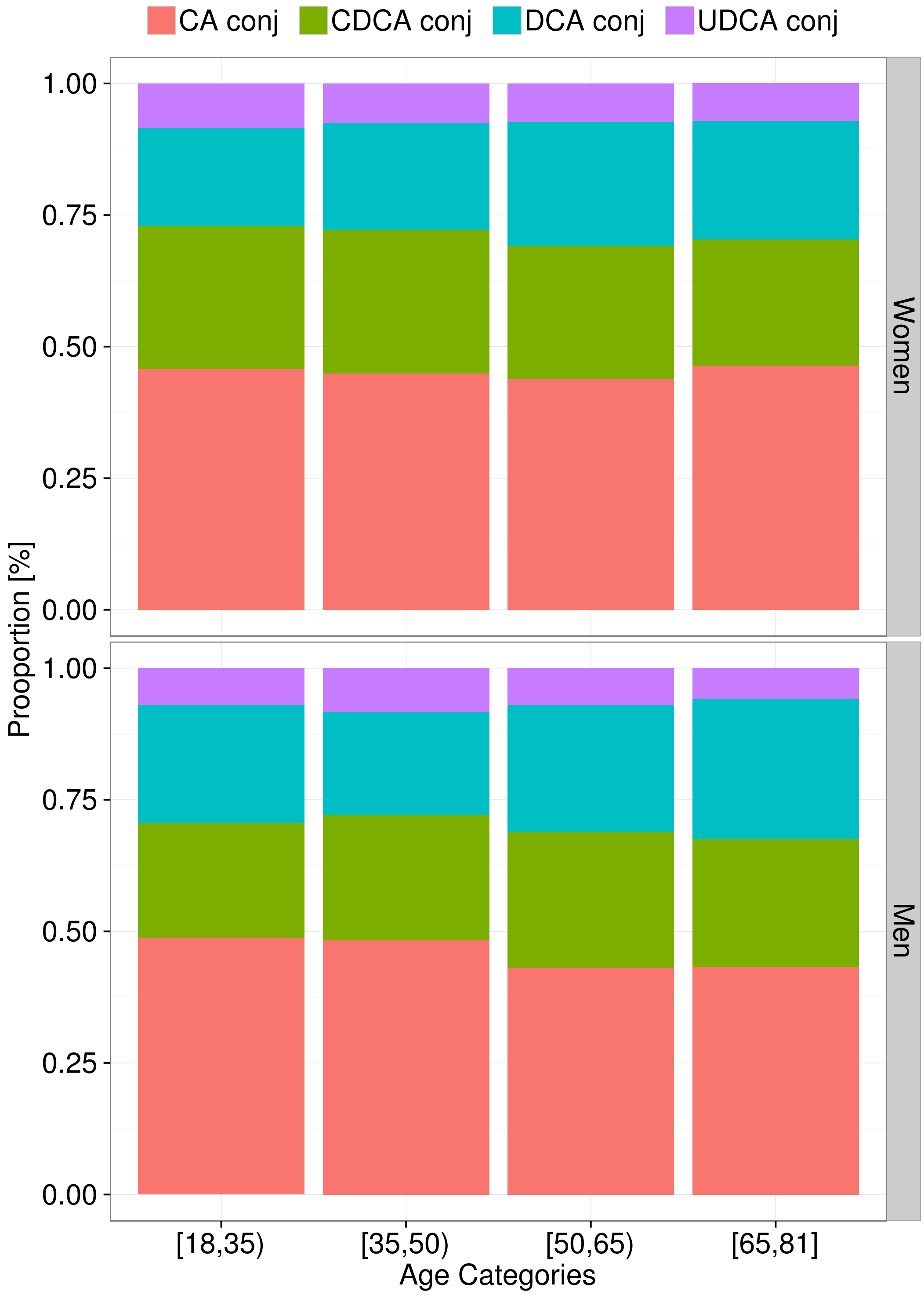

Supplement: S1 Fig — (PDF) [file pone.0153959.s001.pdf]
