# Supplementary material for: Age-Related Changes of Plasma Bile Acid Concentrations in Healthy Adults—Results from the Cross-Sectional KarMeN Study
Source: PLoS One. 2016 Apr 19;11(4):e0153959. doi: 10.1371/journal.pone.0153959 (PMC4836658; doi:10.1371/journal.pone.0153959)
Supplement: S2 Fig — (PDF) [file pone.0153959.s002.pdf]

S2 Figure. Quantile Regression Plots

Plots displaying association of age and sex on plasma BA concentrations. Lines depict the predicted values according to the median regression model for men (light blue) and women (pink).

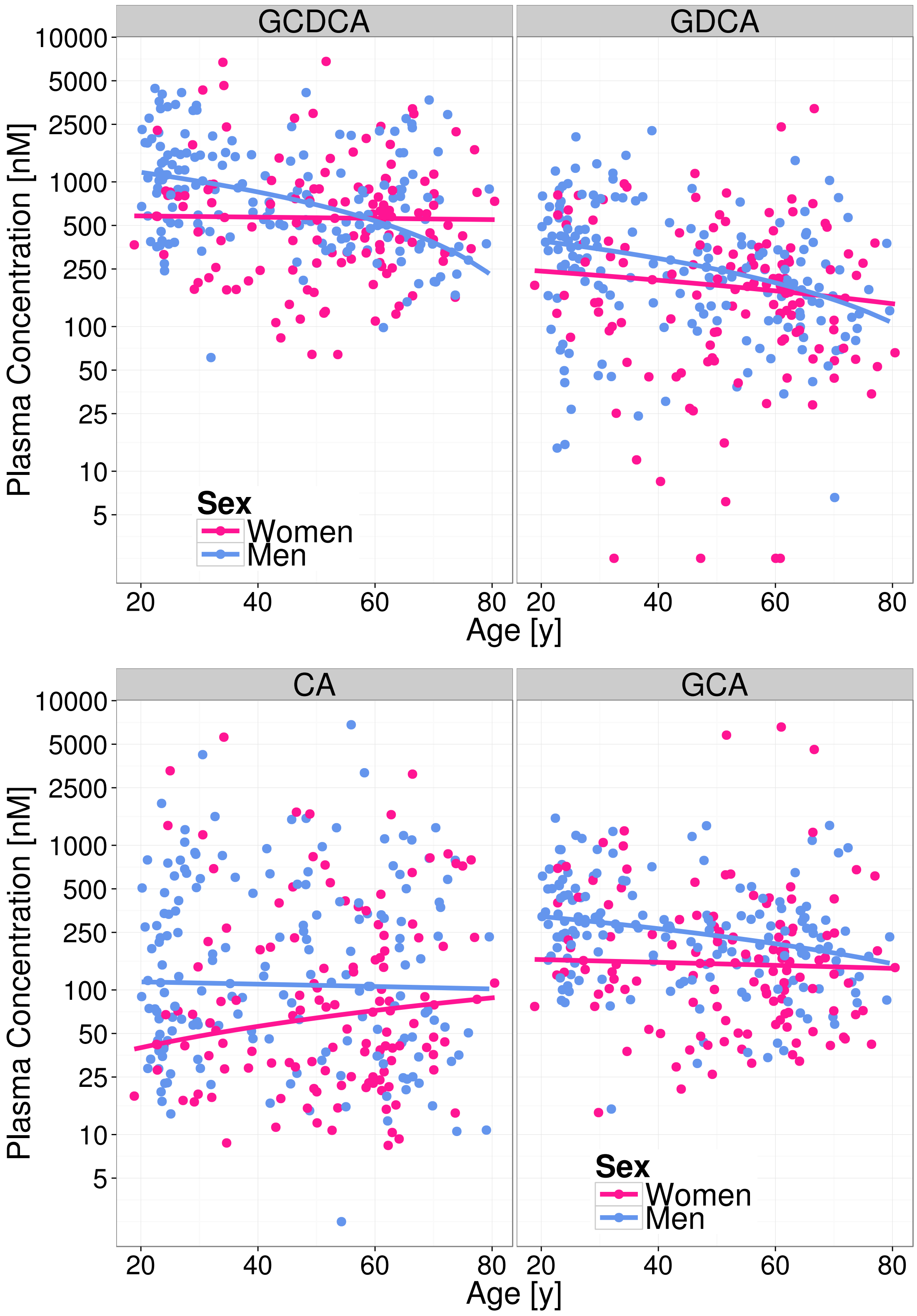

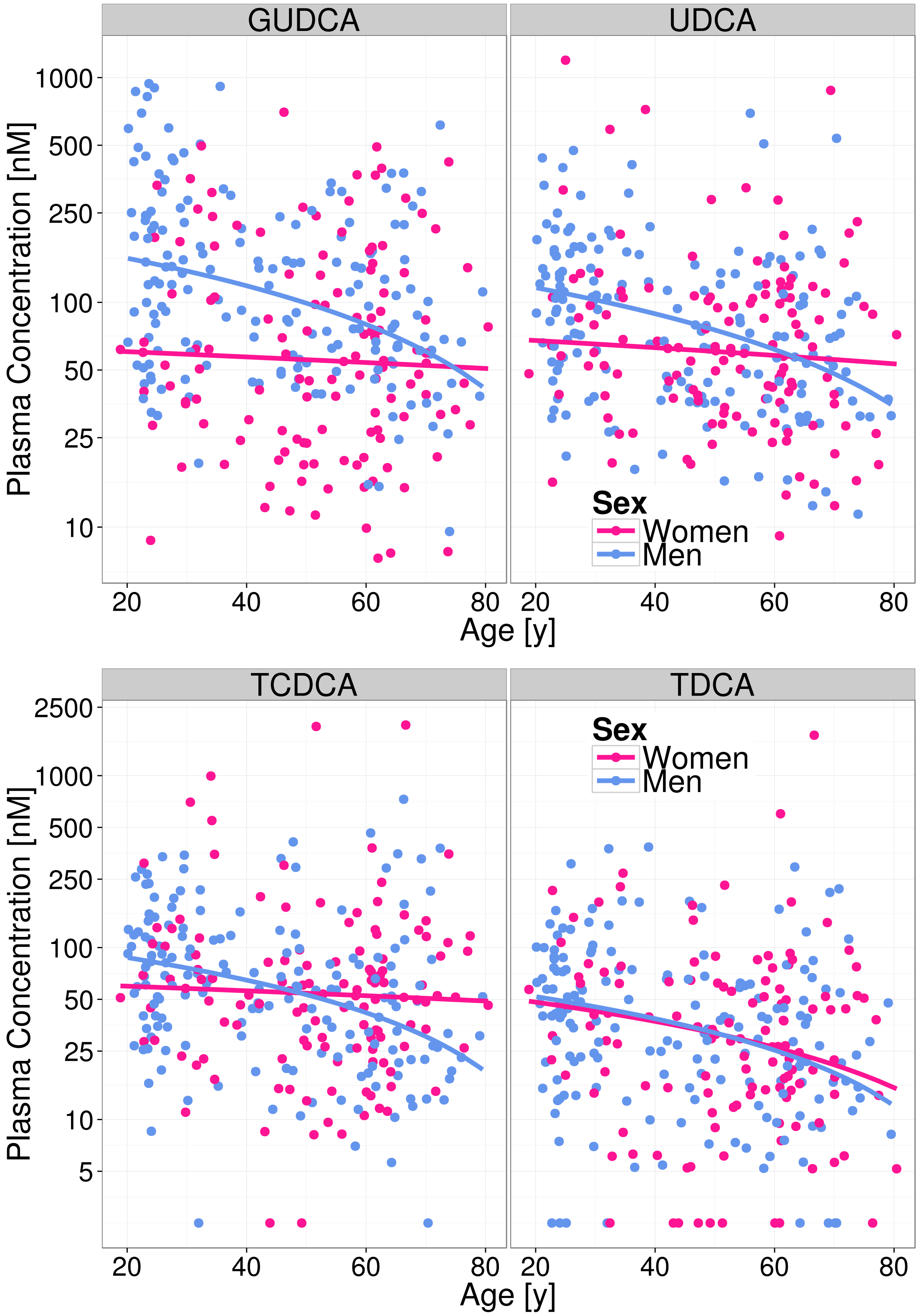
GUDCA
UDCA
TCDCA
TDCA
Plasma Concentration [nM]
Age [y]
Sex
Women
Men
1000
500
250
100
50
25
10
2500
5
20
40
60
80

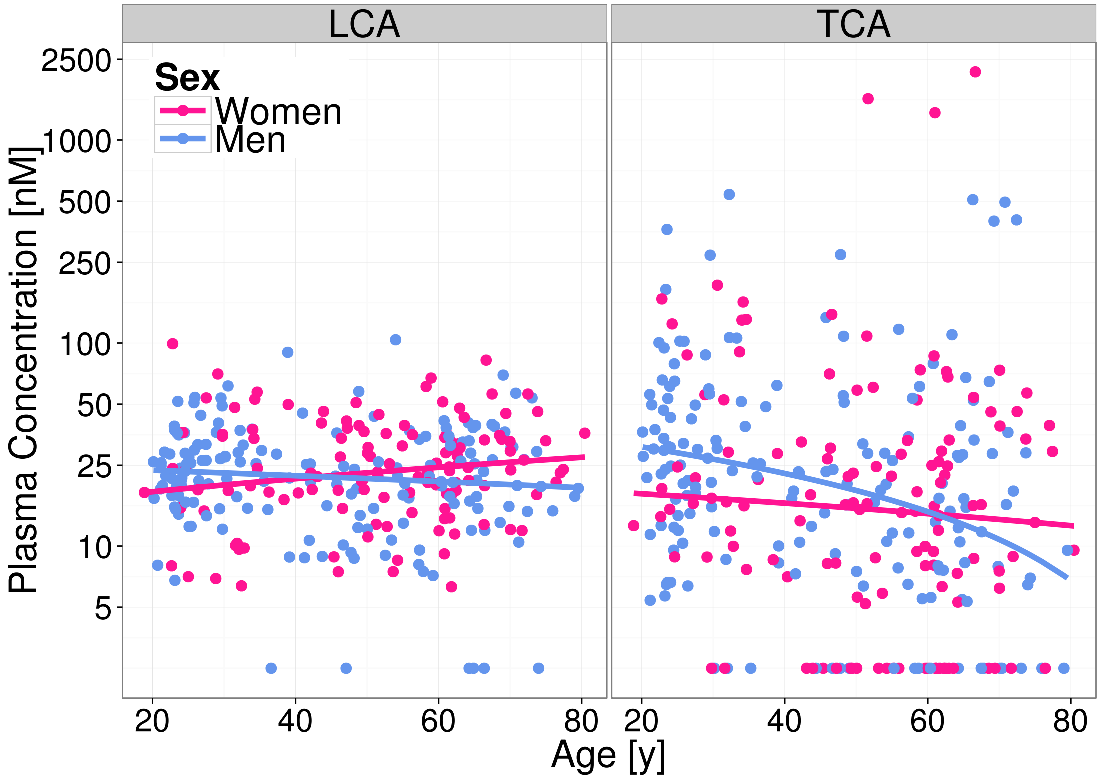
LCA
TCA
Sex
Women
Men
Plasma Concentration [nM]
2500
1000
500
250
100
50
25
10
5
20
40
60
80
Age [y]
