# Supplementary material for: Age-Related Changes of Plasma Bile Acid Concentrations in Healthy Adults—Results from the Cross-Sectional KarMeN Study
Source: PLoS One. 2016 Apr 19;11(4):e0153959. doi: 10.1371/journal.pone.0153959 (PMC4836658; doi:10.1371/journal.pone.0153959)

S3 Figure. Age and Sex association of TG, LDL, HDL and BF%

Association of age and sex with TG, LDL, HDL, BF%, and Energy Fat% within the study population. Lines depict the predicted values according to the median regression model for men (light blue) and women (pink).

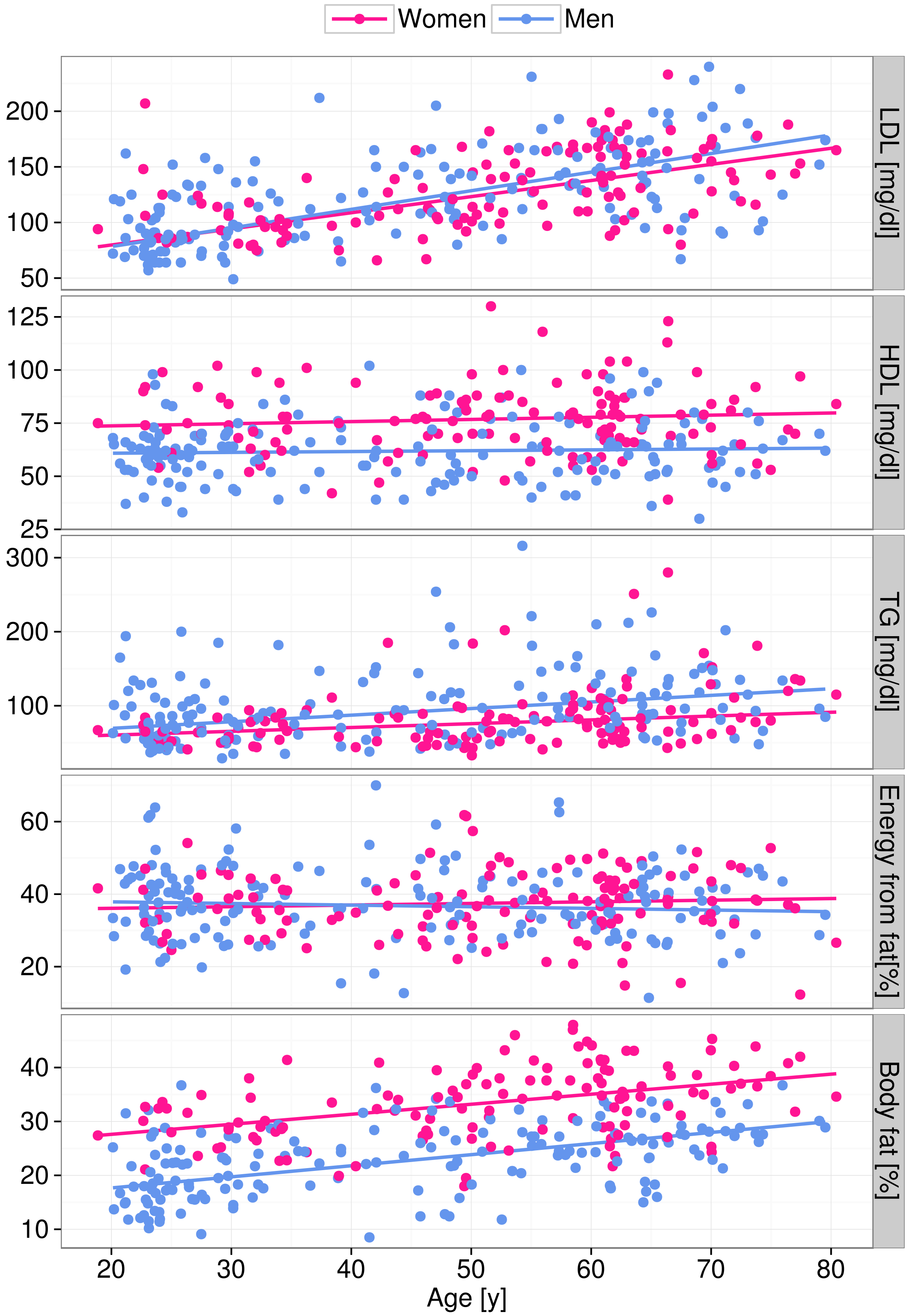

Supplement: S3 Fig — (PDF) [file pone.0153959.s003.pdf]
