# Supplementary material for: Age-Related Changes of Plasma Bile Acid Concentrations in Healthy Adults—Results from the Cross-Sectional KarMeN Study
Source: PLoS One. 2016 Apr 19;11(4):e0153959. doi: 10.1371/journal.pone.0153959 (PMC4836658; doi:10.1371/journal.pone.0153959)
Supplement: S1 Table — (PDF) [file pone.0153959.s004.pdf]

S1 Table. Instrument Settings

Q1 [Da]	Q3 [Da]	ID	DP [Volt]	EP [Volt]	CEP [Volt]	CE [Volt]	CXP [Volt]
407.2	407.2	CA 1	-75	-9	-19	-24	-5.1
391.1	391.1	DCA 1	-80	-10	-22	-12	-6
375.0	375.0	LCA 1	-95	-6.5	-22	-22	-4
391.2	391.2	CDCA 1	-80	-6	-26	-20	-6
482.3	80.1	TLCA 1	-100	-10.5	-24	-100	-9
391.1	391.1	UDCA 1	-85	-10.5	-26	-16	-6
498.2	79.7	TCDCA 1	-95	-7	-20	-104	-6
464.3	74.1	GCA 1	-70	-6.5	-24	-70	-6
448.2	73.9	GCDCA 1	-80	-6.5	-20	-64	-55
498.2	79.9	TDCA 1	-110	-11.5	-30	-106	-6
514.2	79.8	TCA 1	-130	-8	-24	-130	-49
448.2	74.0	GUDCA 1	-65	-6	-20	-60	-54
498.2	80.1	TUDCA 1	-120	-5	-28	-118	-6
448.2	73.9	GDCA 1	-75	-9	-16	-66	-54
411.2	411.2	d4-CA	-85	-7	-16	-14	-4
395.1	395.1	d4-DCA	-70	-7.5	-14	-14	-4
452.3	73.8	d4-GUDCA	-75	-5.5	-20	-58	-47
452.2	74.1	d4-GDCA	-65	-5.5	-20	-54	-55
468.3	73.8	d4-GCA	-80	-6.5	-22	-66	-55
519.3	80.1	d5-TCA	-110	-12	-28	-110	-6

Q1 > Q3 MRM transition for respective BA (ID), declustering potential (DP), entry potential (EP), collision cell entry potential (CEP), collision energy (CE), collision cell exit potential (CXP)
