# Supplementary material for: Age-Related Changes of Plasma Bile Acid Concentrations in Healthy Adults—Results from the Cross-Sectional KarMeN Study
Source: PLoS One. 2016 Apr 19;11(4):e0153959. doi: 10.1371/journal.pone.0153959 (PMC4836658; doi:10.1371/journal.pone.0153959)
Supplement: S2 Table — (PDF) [file pone.0153959.s005.pdf]

S2 Tables. Validation Parameters

A: precision and accuracy for three different plasma concentrations (n=5)

	**accuracy [%]**			**Coefficient of Variation [%]**		
	**25nM**	**100nM**	**1000nM**	**25nM**	**100nM**	**1000nM**
**CA Quanti**	97.7	101.4	102.6	5.3	4.3	2.0
**DCA Quanti**	92.0	98.9	101.8	3.2	4.4	2.6
**LCA Quanti**	93.1	100.7	98.0	12.4	6.9	4.5
**CDCA Quanti**	106.3	107.0	99.5	2.3	3.0	5.7
**TLCA Quanti**	90.7	100.5	103.9	3.0	3.8	2.4
**UDCA Quanti**	95.5	101.2	102.3	6.3	2.8	2.6
**TCDCA Quanti**	97.8	103.4	98.8	4.1	3.6	3.0
**GCA Quanti**	107.8	101.2	97.6	4.0	2.5	2.7
**GCDCA Quanti**	107.3	102.0	100.1	9.4	3.0	6.1
**TDCA Quanti**	100.5	103.3	100.4	3.6	3.3	2.7
**TCA Quanti**	102.4	103.1	101.9	3.4	4.4	1.7
**GUDCA Quanti**	108.8	103.1	107.7	2.1	4.2	1.2
**TUDCA Quanti**	94.0	105.1	101.2	4.2	2.6	2.9
**GDCA Quanti**	114.6	106.5	101.2	5.1	4.5	3.6

B: Precision between batches (n=13) determined by pooled QC samples

	**CA**	**DCA**	**LCA**	**CDCA**	**TLCA**	**UDCA**	**TCDCA**	**GCA**	**GCDCA**	**TDCA**	**TCA**	**GUDCA**	**TUDCA**	**GDCA**
CV (%)	4.6	6.7	10.8	6.6	n.a.	5.9	4.8	5.0	4.9	6.1	13.3	4.7	n.a.	5.2
Mean Concentration	331.2	425.2	19.9	439.4	n.a.	95.6	97.0	324.3	877.6	55.5	53.0	121.8	n.a.	301.6

C: Recovery and matrix effect (n=5) for spiked plasma samples c=250 nM

	**CA**	**DCA**	**LCA**	**CDCA**	**TLCA**	**UDCA**	**TCDCA**	**GCA**	**GCDCA**	**TDCA**	**TCA**	**GUDCA**	**TUDCA**	**GDCA**
Recovery %	87.7	87.4	72.7	99.5	95.6	98.2	88.9	101.8	94.4	91.0	118.1	103.9	101.0	86.3
Matrix Effect %	65.4	99.9	88.2	102.6	100.9	83.6	123.0	124.9	79.4	75.0	92.5	73.9	96.9	110.8
