# Supplementary material for: Age-Related Changes of Plasma Bile Acid Concentrations in Healthy Adults—Results from the Cross-Sectional KarMeN Study
Source: PLoS One. 2016 Apr 19;11(4):e0153959. doi: 10.1371/journal.pone.0153959 (PMC4836658; doi:10.1371/journal.pone.0153959)
Supplement: S3 Table — (PDF) [file pone.0153959.s006.pdf]

Supplemental Table 3 (A-E): Full model quantile regression for TG (A), LDL (B), HDL (C), BF % (D) and Energy Fat % (E). Sex and age are accounted for. Significant results ($P < 0.05$) are highlighted.

$ß_0$ Intercept

$ß_1$ effect of age

$ß_2$ effect of sex

$ß_3$ effect of respective parameter of lipid metabolism

$ß_4$ interaction term age*sex

$ß_5$ interaction age*respective parameter of lipid metabolism

$ß_6$ interaction sex*respective parameter of lipid metabolism

$ß_7$ three way interaction age*sex*respective parameter of lipid metabolism

S3A Table. TG

Bile Acid	β_0	β_1	β_2	β_3	β_4	β_5	β_6	β_7
GCDCA, Estimate	743.717	-15.468	-226.773	2.193	19.348	-0.123	-5.448	0.338
GCDCA, std. Estimate	-0.126	-0.262	-0.113	0.098	0.328	-0.188	-0.244	0.518
GCDCA, p-Value	<1e-04	<1e-04	0.01	0.145	0	0.126	0.027	0.058
DCA, Estimate	380.049	-5.419	-72.447	0.795	3.175	0.031	0.035	-0.001
DCA, std. Estimate	-0.099	-0.218	-0.085	0.084	0.128	0.113	0.004	-0.005
DCA, p-Value	<1e-04	0	0.177	0.404	0.251	0.451	0.982	0.986
GDCA, Estimate	259.604	-5.399	-73.312	0.886	3.585	-0.044	-1.702	0.110
GDCA, std. Estimate	-0.094	-0.245	-0.097	0.106	0.162	-0.182	-0.204	0.449
GDCA, p-Value	<1e-04	0	0.143	0.154	0.247	0.289	0.223	0.175
GCA, Estimate	238.799	-3.280	-110.946	0.379	3.396	0.001	-1.247	0.040
GCA, std. Estimate	-0.082	-0.093	-0.092	0.028	0.096	0.003	-0.093	0.101
GCA, p-Value	<1e-04	0.001	0.001	0.409	0.117	0.964	0.183	0.561
CDCA, Estimate	238.626	-4.501	-114.243	0.012	6.493	-0.004	-0.029	0.003
CDCA, std. Estimate	-0.128	-0.107	-0.080	0.001	0.155	-0.009	-0.002	0.006
CDCA, p-Value	<1e-04	0.004	0.003	0.989	0.007	0.914	0.981	0.979
CA, Estimate	112.077	-0.185	-56.616	-0.005	1.354	-0.026	-0.312	0.049
CA, std. Estimate	-0.155	-0.004	-0.040	-0.000	0.033	-0.057	-0.020	0.107
CA, p-Value	<1e-04	0.896	0.051	0.994	0.446	0.415	0.678	0.386
GUDCA, Estimate	107.141	-2.441	-50.077	0.638	2.487	-0.016	-0.486	0.030
GUDCA, std. Estimate	-0.105	-0.259	-0.155	0.179	0.264	-0.153	-0.136	0.284
GUDCA, p-Value	<1e-04	<1e-04	0	0.001	0	0.087	0.164	0.13
UDCA, Estimate	74.504	-1.456	-18.807	0.143	1.517	0.001	-0.240	0.020
UDCA, std. Estimate	-0.120	-0.197	-0.075	0.051	0.205	0.011	-0.086	0.250
UDCA, p-Value	<1e-04	<1e-04	0.017	0.231	0.007	0.902	0.186	0.154
TCDCA, Estimate	56.240	-1.141	-8.503	0.048	1.377	-0.002	-0.347	0.027
TCDCA, std. Estimate	-0.121	-0.103	-0.022	0.012	0.124	-0.019	-0.083	0.222
TCDCA, p-Value	<1e-04	<1e-04	0.259	0.666	0.003	0.723	0.019	0.07
TDCA, Estimate	34.576	-0.693	-8.620	0.143	0.667	-0.005	-0.319	0.019
TDCA, std. Estimate	-0.103	-0.101	-0.037	0.055	0.097	-0.064	-0.123	0.255
TDCA, p-Value	<1e-04	<1e-04	0.206	0.073	0.072	0.338	0.029	0.025
LCA, Estimate	21.487	-0.066	0.444	0.000	0.218	0.001	0.014	0.001
LCA, std. Estimate	-0.151	-0.074	0.014	0.001	0.243	0.057	0.042	0.105
LCA, p-Value	<1e-04	0.349	0.866	0.984	0.185	0.684	0.834	0.796
TCA, Estimate	19.370	-0.413	-6.210	0.023	0.388	0.001	-0.108	0.000
TCA, std. Estimate	-0.099	-0.038	-0.017	0.006	0.036	0.006	-0.026	0.004
TCA, p-Value	<1e-04	<1e-04	0.043	0.634	0.06	0.792	0.187	0.943

S3B Table. LDL

Bile Acid	β_0	β_1	β_2	β_3	β_4	β_5	β_6	β_7
GCDCA, Estimate	678.301	-12.293	-102.308	-1.996	8.783	0.156	1.905	-0.010
GCDCA, std. Estimate	-0.158	-0.208	-0.051	-0.076	0.149	0.204	0.073	-0.013
GCDCA, p-Value	<1e-04	0.001	0.275	0.187	0.097	0.131	0.428	0.951
DCA, Estimate	380.992	-3.154	-57.942	-1.007	2.465	0.026	1.012	-0.121
DCA, std. Estimate	-0.098	-0.127	-0.068	-0.091	0.099	0.080	0.092	-0.375
DCA, p-Value	<1e-04	0.162	0.322	0.143	0.446	0.53	0.572	0.271
GDCA, Estimate	244.368	-4.298	-21.832	-0.627	1.001	0.034	2.116	-0.116
GDCA, std. Estimate	-0.114	-0.195	-0.029	-0.064	0.045	0.118	0.216	-0.405
GDCA, p-Value	<1e-04	0	0.583	0.246	0.668	0.249	0.088	0.098
GCA, Estimate	230.311	-3.044	-85.500	-0.057	1.908	0.026	0.257	-0.004
GCA, std. Estimate	-0.089	-0.086	-0.071	-0.004	0.054	0.056	0.016	-0.010
GCA, p-Value	<1e-04	0	0.001	0.911	0.283	0.36	0.761	0.939
CDCA, Estimate	225.259	-2.966	-99.820	-0.649	5.255	0.026	0.559	-0.033
CDCA, std. Estimate	-0.138	-0.071	-0.070	-0.035	0.125	0.048	0.030	-0.061
CDCA, p-Value	<1e-04	0.056	0.01	0.409	0.024	0.578	0.598	0.708
CA, Estimate	107.880	-0.154	-42.054	-0.175	1.110	0.007	-0.023	-0.007
CA, std. Estimate	-0.158	-0.004	-0.030	-0.010	0.027	0.012	-0.001	-0.014
CA, p-Value	0	0.923	0.157	0.796	0.548	0.865	0.979	0.873
GUDCA, Estimate	92.037	-1.625	-41.561	-0.069	1.050	0.017	0.227	0.001
GUDCA, std. Estimate	-0.152	-0.172	-0.129	-0.017	0.111	0.140	0.054	0.012
GUDCA, p-Value	<1e-04	0.026	0.007	0.839	0.205	0.371	0.649	0.962
UDCA, Estimate	75.218	-1.346	-15.244	-0.022	1.447	0.006	-0.220	-0.005
UDCA, std. Estimate	-0.117	-0.182	-0.060	-0.007	0.196	0.061	-0.067	-0.050
UDCA, p-Value	<1e-04	0.001	0.127	0.924	0.047	0.597	0.562	0.844
TCDCA, Estimate	51.021	-0.971	3.368	-0.037	0.662	0.010	0.005	0.005
TCDCA, std. Estimate	-0.135	-0.088	0.009	-0.007	0.060	0.071	0.001	0.037
TCDCA, p-Value	<1e-04	0.001	0.664	0.783	0.23	0.124	0.982	0.74
TDCA, Estimate	31.621	-0.548	-0.677	-0.071	0.092	0.005	0.046	-0.000
TDCA, std. Estimate	-0.116	-0.080	-0.003	-0.023	0.013	0.055	0.015	-0.002
TDCA, p-Value	<1e-04	0.01	0.914	0.451	0.805	0.353	0.769	0.987
LCA, Estimate	22.512	0.011	0.428	-0.063	0.155	0.000	0.040	-0.002
LCA, std. Estimate	-0.117	0.012	0.014	-0.158	0.173	0.004	0.101	-0.148
LCA, p-Value	<1e-04	0.892	0.907	0.072	0.432	0.981	0.673	0.767
TCA, Estimate	17.990	-0.339	-2.569	-0.004	0.222	0.003	0.032	-0.002
TCA, std. Estimate	-0.102	-0.031	-0.007	-0.001	0.021	0.022	0.007	-0.017
TCA, p-Value	<1e-04	0.009	0.41	0.956	0.34	0.445	0.74	0.765

S3C Table. HDL

Bile Acid	β_0	β_1	β_2	β_3	β_4	β_5	β_6	β_7
GCDCA, Estimate	739.229	-12.531	-186.151	-1.329	16.682	0.372	3.450	-0.699
GCDCA, std. Estimate	-0.128	-0.213	-0.092	-0.022	0.283	0.211	0.057	-0.396
GCDCA, p-Value	<1e-04	0	0.065	0.792	0.007	0.244	0.607	0.107
DCA, Estimate	413.826	-4.983	-101.670	3.117	4.110	-0.152	-3.548	0.100
DCA, std. Estimate	-0.059	-0.200	-0.120	0.123	0.165	-0.204	-0.140	0.135
DCA, p-Value	<1e-04	0.001	0.038	0.089	0.191	0.188	0.195	0.567
GDCA, Estimate	259.161	-4.525	-79.126	0.697	4.757	0.058	1.968	-0.319
GDCA, std. Estimate	-0.094	-0.205	-0.105	0.031	0.216	0.087	0.087	-0.484
GDCA, p-Value	<1e-04	0.002	0.047	0.651	0.032	0.525	0.448	0.016
GCA, Estimate	237.170	-3.082	-82.730	-0.466	3.228	-0.013	0.356	-0.047
GCA, std. Estimate	-0.084	-0.087	-0.068	-0.013	0.091	-0.012	0.010	-0.044
GCA, p-Value	<1e-04	0.015	0.002	0.765	0.066	0.893	0.882	0.711
CDCA, Estimate	247.127	-5.393	-110.781	0.436	7.414	-0.070	-1.158	0.064
CDCA, std. Estimate	-0.122	-0.129	-0.077	0.010	0.177	-0.056	-0.027	0.051
CDCA, p-Value	<1e-04	0	0.004	0.757	0.002	0.444	0.527	0.624
CA, Estimate	104.907	0.312	-43.806	-1.447	0.494	0.049	1.516	-0.050
CA, std. Estimate	-0.160	0.008	-0.031	-0.034	0.012	0.039	0.036	-0.040
CA, p-Value	0	0.827	0.152	0.33	0.776	0.535	0.357	0.577
GUDCA, Estimate	100.755	-1.587	-40.985	-0.587	1.240	0.040	-0.020	-0.019
GUDCA, std. Estimate	-0.125	-0.168	-0.127	-0.061	0.131	0.143	-0.002	-0.069
GUDCA, p-Value	<1e-04	0.008	0.014	0.333	0.169	0.269	0.983	0.702
UDCA, Estimate	74.846	-1.506	-14.042	-0.249	1.538	-0.024	0.318	-0.002
UDCA, std. Estimate	-0.118	-0.204	-0.056	-0.033	0.208	-0.110	0.042	-0.009
UDCA, p-Value	<1e-04	<1e-04	0.204	0.434	0.065	0.239	0.538	0.957
TCDCA, Estimate	58.106	-1.251	-6.493	0.149	1.194	-0.017	0.117	0.003
TCDCA, std. Estimate	-0.116	-0.113	-0.017	0.013	0.108	-0.050	0.010	0.010
TCDCA, p-Value	<1e-04	0.001	0.507	0.741	0.045	0.542	0.842	0.924
TDCA, Estimate	38.347	-0.702	-9.725	0.490	0.484	-0.003	-0.266	-0.015
TDCA, std. Estimate	-0.087	-0.103	-0.042	0.070	0.071	-0.013	-0.038	-0.071
TDCA, p-Value	<1e-04	0.004	0.156	0.066	0.217	0.878	0.498	0.578
LCA, Estimate	21.953	-0.120	1.982	0.036	0.308	-0.007	0.053	-0.006
LCA, std. Estimate	-0.136	-0.133	0.065	0.040	0.344	-0.248	0.058	-0.206
LCA, p-Value	<1e-04	0.156	0.32	0.677	0.015	0.254	0.707	0.561
TCA, Estimate	20.394	-0.450	-5.153	0.101	0.385	-0.014	-0.063	0.011
TCA, std. Estimate	-0.096	-0.042	-0.014	0.009	0.036	-0.043	-0.006	0.033
TCA, p-Value	<1e-04	<1e-04	0.105	0.446	0.087	0.228	0.773	0.458

S3D Table. Body Fat %

Bile Acid	β_0	β_1	β_2	β_3	β_4	β_5	β_6	β_7
GCDCA, Estimate	679.420	-11.977	-50.618	-11.105	13.148	0.198	4.379	-0.619
GCDCA, std. Estimate	-0.158	-0.203	-0.025	-0.092	0.223	0.056	0.036	-0.175
GCDCA, p-Value	<1e-04	0.001	0.572	0.338	0.007	0.755	0.787	0.523
DCA, Estimate	399.262	-4.489	-153.037	-0.188	-0.069	-0.033	6.211	0.265
DCA, std. Estimate	-0.076	-0.180	-0.180	-0.004	-0.003	-0.022	0.121	0.177
DCA, p-Value	<1e-04	0.058	0.03	0.976	0.985	0.911	0.47	0.563
GDCA, Estimate	243.481	-4.289	-40.952	-4.167	2.143	0.041	5.154	-0.084
GDCA, std. Estimate	-0.115	-0.194	-0.054	-0.092	0.097	0.031	0.114	-0.063
GDCA, p-Value	<1e-04	0	0.36	0.21	0.466	0.834	0.398	0.831
GCA, Estimate	241.993	-3.071	-59.439	0.932	2.449	0.028	-6.647	0.063
GCA, std. Estimate	-0.080	-0.087	-0.049	0.013	0.069	0.013	-0.091	0.030
GCA, p-Value	<1e-04	0.002	0.047	0.668	0.157	0.85	0.128	0.815
CDCA, Estimate	246.143	-4.475	-104.282	2.498	7.410	0.044	-5.750	-0.139
CDCA, std. Estimate	-0.123	-0.107	-0.073	0.029	0.177	0.018	-0.067	-0.055
CDCA, p-Value	<1e-04	0.018	0.038	0.603	0.001	0.855	0.313	0.677
CA, Estimate	106.609	-0.308	-36.207	-1.686	1.295	-0.120	0.545	0.126
CA, std. Estimate	-0.159	-0.007	-0.026	-0.020	0.031	-0.048	0.006	0.051
CA, p-Value	0	0.847	0.304	0.722	0.521	0.575	0.916	0.6
GUDCA, Estimate	106.827	-2.014	-57.602	1.069	1.822	0.046	0.119	-0.087
GUDCA, std. Estimate	-0.106	-0.213	-0.179	0.055	0.193	0.081	0.006	-0.153
GUDCA, p-Value	<1e-04	0.002	0	0.537	0.03	0.615	0.956	0.422
UDCA, Estimate	82.296	-1.457	-22.956	1.384	0.957	0.044	-1.589	0.032
UDCA, std. Estimate	-0.089	-0.197	-0.091	0.091	0.130	0.098	-0.105	0.073
UDCA, p-Value	<1e-04	0	0.028	0.043	0.194	0.385	0.202	0.737
TCDCA, Estimate	54.799	-1.041	0.256	-0.482	0.798	0.009	-0.215	0.027
TCDCA, std. Estimate	-0.125	-0.094	0.001	-0.021	0.072	0.013	-0.009	0.041
TCDCA, p-Value	<1e-04	<1e-04	0.977	0.555	0.161	0.84	0.868	0.801
TDCA, Estimate	31.691	-0.485	-0.611	-0.548	-0.197	-0.008	0.797	0.022
TDCA, std. Estimate	-0.115	-0.071	-0.003	-0.039	-0.029	-0.020	0.057	0.054
TDCA, p-Value	<1e-04	0.046	0.924	0.319	0.583	0.797	0.359	0.714
LCA, Estimate	22.939	-0.068	-0.806	0.370	0.254	0.005	-0.214	-0.018
LCA, std. Estimate	-0.103	-0.076	-0.026	0.201	0.283	0.096	-0.116	-0.325
LCA, p-Value	<1e-04	0.423	0.8	0.011	0.319	0.615	0.638	0.625
TCA, Estimate	20.248	-0.426	-2.417	0.137	0.430	-0.006	-0.617	0.007
TCA, std. Estimate	-0.096	-0.040	-0.007	0.006	0.040	-0.010	-0.028	0.012
TCA, p-Value	<1e-04	0	0.445	0.63	0.027	0.706	0.168	0.804

S3E Table. Energy Fat %

Bile Acid	β_0	β_1	β_2	β_3	β_4	β_5	β_6	β_7
GCDCA, Estimate	732.740	-15.057	-152.840	0.408	14.331	-0.114	-2.471	0.232
GCDCA, std. Estimate	-0.131	-0.255	-0.076	0.004	0.243	-0.037	-0.024	0.076
GCDCA, p-Value	<1e-04	<1e-04	0.093	0.933	0.001	0.748	0.772	0.661
DCA, Estimate	392.227	-4.421	-71.564	2.635	3.167	-0.106	0.997	0.276
DCA, std. Estimate	-0.084	-0.178	-0.084	0.060	0.127	-0.082	0.023	0.213
DCA, p-Value	<1e-04	0.008	0.12	0.535	0.188	0.515	0.865	0.297
GDCA, Estimate	260.449	-4.555	-75.005	0.097	4.607	-0.136	3.714	0.191
GDCA, std. Estimate	-0.093	-0.206	-0.100	0.002	0.209	-0.118	0.095	0.167
GDCA, p-Value	<1e-04	<1e-04	0.042	0.97	0.043	0.347	0.423	0.507
GCA, Estimate	243.963	-2.942	-96.263	1.230	2.364	-0.039	-1.655	0.013
GCA, std. Estimate	-0.078	-0.083	-0.080	0.020	0.067	-0.021	-0.026	0.007
GCA, p-Value	<1e-04	0.001	0.001	0.435	0.173	0.732	0.564	0.949
CDCA, Estimate	241.453	-4.639	-113.118	1.680	7.342	-0.096	-0.446	0.205
CDCA, std. Estimate	-0.126	-0.111	-0.079	0.023	0.175	-0.044	-0.006	0.094
CDCA, p-Value	<1e-04	0	0.002	0.483	<1e-04	0.555	0.892	0.315
CA, Estimate	108.608	-0.243	-43.328	-0.068	1.148	0.006	0.259	0.010
CA, std. Estimate	-0.157	-0.006	-0.031	-0.001	0.028	0.003	0.004	0.005
CA, p-Value	<1e-04	0.868	0.127	0.975	0.478	0.966	0.919	0.953
GUDCA, Estimate	105.773	-1.892	-50.046	-0.946	1.852	-0.022	1.732	0.017
GUDCA, std. Estimate	-0.109	-0.201	-0.155	-0.056	0.196	-0.044	0.103	0.035
GUDCA, p-Value	<1e-04	0.001	0	0.311	0.006	0.761	0.167	0.847
UDCA, Estimate	78.783	-1.381	-13.055	-0.435	1.193	-0.036	1.784	0.046
UDCA, std. Estimate	-0.103	-0.187	-0.052	-0.033	0.161	-0.095	0.136	0.119
UDCA, p-Value	<1e-04	<1e-04	0.081	0.45	0.039	0.296	0.085	0.473
TCDCA, Estimate	55.558	-1.027	0.064	0.007	0.806	-0.045	-0.190	0.046
TCDCA, std. Estimate	-0.123	-0.093	0.000	0.000	0.073	-0.077	-0.010	0.080
TCDCA, p-Value	<1e-04	0	0.993	0.988	0.062	0.125	0.834	0.452
TDCA, Estimate	35.361	-0.593	-2.324	0.118	0.093	-0.021	-0.026	0.035
TDCA, std. Estimate	-0.100	-0.087	-0.010	0.010	0.014	-0.060	-0.002	0.098
TDCA, p-Value	<1e-04	0.001	0.7	0.783	0.779	0.353	0.971	0.42
LCA, Estimate	22.662	-0.015	-0.111	0.124	0.155	0.008	-0.166	-0.008
LCA, std. Estimate	-0.112	-0.017	-0.004	0.078	0.173	0.176	-0.104	-0.178
LCA, p-Value	<1e-04	0.837	0.96	0.4	0.276	0.288	0.486	0.568
TCA, Estimate	19.201	-0.343	-3.458	-0.017	0.257	-0.012	-0.248	0.021
TCA, std. Estimate	-0.099	-0.032	-0.009	-0.001	0.024	-0.021	-0.013	0.037
TCA, p-Value	<1e-04	<1e-04	0.212	0.898	0.132	0.141	0.413	0.306
