# Supplementary material for: Age-Related Changes of Plasma Bile Acid Concentrations in Healthy Adults—Results from the Cross-Sectional KarMeN Study
Source: PLoS One. 2016 Apr 19;11(4):e0153959. doi: 10.1371/journal.pone.0153959 (PMC4836658; doi:10.1371/journal.pone.0153959)
Supplement: S4 Table — (DOCX) [file pone.0153959.s007.docx]

|  | **Male n = 172** | | | | | | | **Female n = 128** | | | | | | **All n = 300** | | | | | |
| --- | --- | --- | --- | --- | --- | --- | --- | --- | --- | --- | --- | --- | --- | --- | --- | --- | --- | --- | --- |
|  | **Median** | | **Q1** | **Q3** | **Min** | **Max** | **Spearman Cor.** | **Median** | **Q1** | **Q3** | **Min** | **Max** | **Spearman Cor.** | **Median** | **Q1** | **Q3** | **Min** | **Max** | **Spearman Cor.** |
| **Fasting Glucose (mg/dl)** | 86 | 81 | | 91 | 69 | 114 | 0.510 | 85 | 81 | 89 | 69 | 116 | 0.396 | 85 | 81 | 90 | 69 | 116 | 0.438 |
| **Fiber intake**  **(g/kg)*** | 0.33 | 0.24 | | 0.46 | 0.03 | 1.27 | -0.083 | 0.34 | 0.25 | 0.48 | 0.10 | 0.86 | 0.081 | 0.33 | 0.25 | 0.47 | 0.03 | 1.27 | -0.010 |

S4 Table. Additional data of the participants stratified by sex.

*fiber intake calculated in g / kg body weight

Data presented is displaying the respective median values, the 25^th^ percentile (Q1), the 75^th^ percentile (Q3), the minimum (Min) and maximum (Max) values. Correlation of data with age is indicated by Spearman correlation (r_S_).
